# Is single reading with computer-aided detection (CAD) as good as double reading in mammography screening? A systematic review

**DOI:** 10.1186/1471-2342-12-22

**Published:** 2012-07-24

**Authors:** Edward Azavedo, Sophia Zackrisson, Ingegerd Mejàre, Marianne Heibert Arnlind

**Affiliations:** 1 Department of Diagnostic Radiology, Karolinska Institutet, Stockholm, Sweden; 2 Department of Clinical Sciences in Malmö, Diagnostic Radiology, Lund University, Skåne University Hospital Malmö, Malmö, SE-205 02, Sweden; 3 Swedish Council on Health Technology Assessment (SBU), Stockholm, Sweden; 4 LIME/MMC, Karolinska Institutet, Stockholm, Sweden

**Keywords:** CAD, Mammography, Screening, Breast, Cancer, Single reading, Double reading

## Abstract

**Background:**

In accordance with European guidelines, mammography screening comprises independent readings by two breast radiologists (double reading). CAD (computer-aided detection) has been suggested to complement or replace one of the two readers (single reading + CAD).

The *aim* of this systematic review is to address the following question: Is the reading of mammographic x-ray images by a single breast radiologist together with CAD at least as accurate as double reading?

**Methods:**

The electronic literature search included the databases Pub Med, EMBASE and The Cochrane Library. Two independent reviewers assessed abstracts and full-text articles.

**Results:**

1049 abstracts were identified, of which 996 were excluded with reference to inclusion and exclusion criteria; 53 full-text articles were assessed for eligibility. Finally, four articles were included in the qualitative analysis, and one in a GRADE synthesis.

**Conclusions:**

The scientific evidence is insufficient to determine whether the accuracy of *single reading + CAD* is at least equivalent to that obtained in standard practice, i.e. *double reading* where two breast radiologists independently read the mammographic images.

## Background

Following reports from Swedish randomized trials [1-4], breast cancer screening programs with mammography have been established in recent decades in many countries [5]. The age range of women invited to screening varies between countries. The Swedish National Board of Health and Welfare recommends mammography screening at regular intervals to all women between 40 and 74 years. The initial results from the randomized trials, showing a reduction in mortality in breast cancer, have been confirmed by long-term follow-up [6,7] Similar results have been obtained in established population-based service screening programs [8,9]. However, the pros and cons of mammography screening and how the results should be interpreted [10] are still matters for debate.

Besides the primary aim of detecting breast cancers in screening programs, it is important that recall rates are kept as low as possible without impairing detection rates. In this respect, the recommended recall rate in Sweden and in the rest of Europe should not exceed five per cent [11]. The reasons for recall are several, such as suspicious findings suggesting malignancy, indeterminate findings that need further work-up, and occasionally for technical reasons or if the woman reports clinical symptoms at the time of the screening examination.

**Figure 1:**
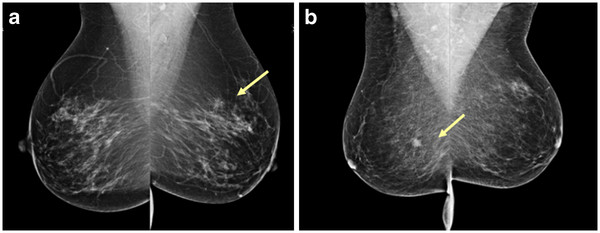
**Figure**1**a shows a rather hard to detect breast cancer in the left breast (arrow); the right breast is normal.** Figure 1b shows an easily detected cancer in the right breast (arrow); the left breast is normal.

As the radiological image of breast tissue is complex, mammograms need to be interpreted by highly specialized radiologists. Figure 1 shows an example of mammography images.

Factors that affect the ability to detect a breast cancer (sensitivity) are e.g. the prevalence of breast cancer in the target population, dense breast tissue, the frequency of tumours with subtle mammographic signs, and suboptimal technical quality. These factors, combined with high daily volumes (each Swedish screening centre usually screens more than 20,000 women annually), makes accurate screening a challenging task. Sensitivity levels of 70–85% and specificity levels of 82–98% at mammography screening have been reported [5]. In order to maintain high sensitivity and specificity, resulting in high cancer detection rates and low false-positive rates, Swedish and European guidelines recommend double reading, i.e. that the breast images are reviewed by two specially trained radiologists (breast radiologists). Double reading has been shown to increase cancer detection rates by 5–17% [12].

Computer-aided detection (CAD) is a computerized method for analysing images in mammography screening. Although the method has existed for approximately 10 years, its contribution to routine screening is still debatable [13-15]. The program used in CAD identifies and marks areas which the software identifies as abnormal breast tissue. The CAD program is not intended to be the sole method for analysing mammography images. Rather, it is designed to alert the radiologist to possibly suspicious areas. Hence, a radiologist must interpret and make a decision to act upon (accept or dismiss) each CAD mark. On average, each screening examination generates two false positive marks; CAD gives 400 false positive marks for each true positive mark [16].

Lack of an adequate number of trained breast radiologists has led to a growing interest in computerized analysis of mammography images. There has been a discussion as to whether CAD in conjunction with mammography screening could replace one of the breast radiologists. A prerequisite would be that diagnostic accuracy and patient benefit are at least equivalent to what is achieved when the mammographic images are read by two breast radiologists. Another important prerequisite is that not too many women need to be recalled for further diagnostic work-up.

The value of CAD in mammography screening has been questioned in earlier reviews [17,18]. The literature is scarce on studies performed in authentic screening situations. As the performance of CAD systems has improved considerably, it was considered appropriate to reassess the performance of CAD in population-based screening programs.

This review is part of a comprehensive systematic review, published in Swedish by SBU (Swedish Council on Health Technology Assessment), of computer-aided detection (CAD) as a diagnostic method in mammography screening [19]. SBU is an independent government agency for the critical evaluation of methods for preventing, diagnosing and treating health problems.

The objective of the present is systematic review is to address the following question: Is the reading of mammographic images by a single breast radiologist plus CAD at least as accurate as readings by two breast radiologists (current practice) in terms of:

· sensitivity (probability that a person with the disease has a positive test result);

· specificity (probability that a healthy person has a negative test result);

· cancer detection rate (number of cancer cases detected per 1,000 women examined);

· recall rate (proportion of women who are recalled for further investigation); and

· cost-effectiveness?

## Methods

### CAD (Computer-aided detection)

CAD research has been developed over the past two decades. CAD was first applied to digitized (scanned) screen-film mammograms (SFM). The introduction of full-field digital mammography (FFDM) has led to intensified efforts to optimise the method. CAD makes a computerized analysis of mammograms and identifies areas that need to be reviewed. The precise algorithms used by different CAD suppliers are still a commercial secret and are not further reviewed here. Two types of marks are generally used: one for microcalcifications and the other for other mammographic features such as density, mass and distortion. The systems can be adjusted to yield very high sensitivity but at the cost of specificity, generating a high rate of false positive marks.

According to a recent review, the sensitivity of CAD for microcalcifications representing malignancies is 98–99% [16]. However, only 15–20% of detected cancers present as microcalcifications on screening mammograms [20]. The same review reports that the sensitivity of CAD for other mammographic features representing malignancies ranges from 89 to 75%, in some cases down to 50%.

It has been assumed that CAD will be used increasingly with the transition from analogue to digital mammography. The reproducibility of CAD prompts in FFDM is expected to be more consistent than with scanned mammograms. The primary inclusion criterion in this review was CAD on FFDMs. However, when prospective studies based on FFDM could not be found, scanned analogue images were accepted.

### Literature search and selection of articles

The electronic literature search included the databases PubMed, EMBASE, and The Cochrane Library from 1950 to November 2011. All Western European languages were accepted. The Mesh terms were: Breast neoplasms, Breast, Mammography, Breast (TW), Mammography (TW) AND Computer aided detection (TW) AND Computer aided diagnosis (TW) AND Cad (TW), and Economic aspects. The complete search strategy can be provided on request.

**Figure 2:**
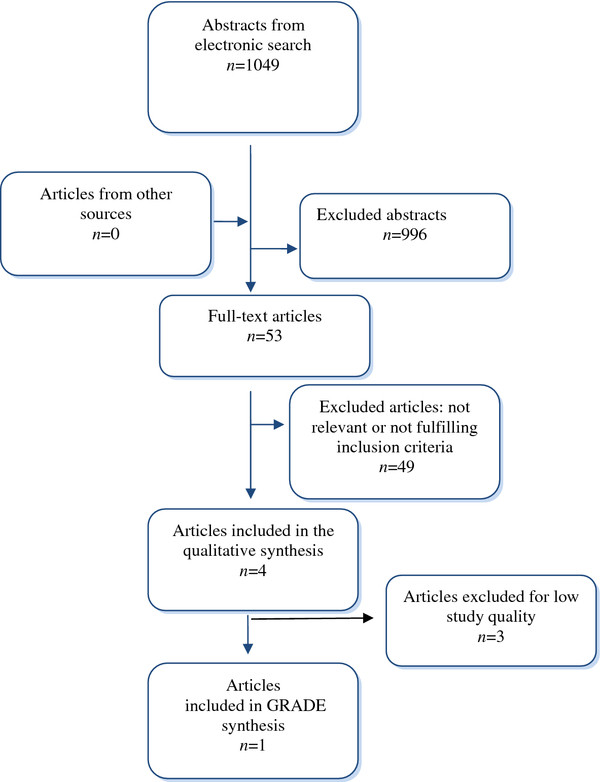
Flow chart of the search strategy.

The electronic searches yielded 1049 abstracts (Figure 2). Two reviewers (EA and SZ) read the abstracts independently. An article was read in full text if at least one of the two reviewers considered an abstract to be potentially relevant. Hand search and grey literature did not result in any additional articles. The pre-specified inclusion/exclusion criteria are given below. Altogether, 53 articles were read in full text and assessed independently by the same two reviewers using the QUADAS tool [21]. Of the 53 articles, 49 did not fulfil the inclusion criteria and were excluded from further analysis. A list of excluded articles with the main reason for exclusion is available on request. 

PICO elements were used to describe the population, index test, reference test and outcome:

P – Population: women, 40–74 years old, participating in mammography screening

I – Intervention (index test): CAD + one breast radiologist (single reading)

C – Control (reference test): reading by two independent radiologists (double reading)

O – Outcome: sensitivity, specificity, cancer detection rate and recall rate

The inclusion criteria were:

· population-based screening

· ≥5,000 women included

· study setting corresponding to Swedish conditions

· follow-up time ≥ 12 months

· mammography readings with one breast radiologist + CAD compared with readings by two breast radiologists.

### Assessment of diagnostic accuracy

The diagnostic accuracy (validity) of a test (index test) requires a reference standard (reference test) for comparison. Two index tests were used here: 1) CAD + single reading, and 2) double reading. The reference standard should reflect the reality as closely as possible and the ideal gold standard is histopathological verification. However, biopsying all individuals is not feasible when screening an asymptomatic population. The reference standard in this review was biopsy of suspected cases or follow-up. The ultimate outcome was survival. Because no randomized controlled trials have been performed to document changes in survival following the use of double reading compared to single reading with CAD, surrogate outcomes such as cancer detection rate and recall rate are used. The main outcome measures are sensitivity and specificity. Sensitivity is the number of true positive tests divided by the total number of true cancer cases. Specificity is the number of true negative tests divided by the total number of healthy breast cases. In addition, cost-effectiveness has been considered.

### Rating quality of individual studies

The quality of each included study was rated high, moderate or low according to pre-specified criteria given in Table 1.

**Table 1:** **Criteria of high, moderate and low study quality, mainly according to QUADAS**[21]

*High: small risk of bias*	Prospective study design. Particular emphasis on the following:
	● adequately described patients constituting a representative and clinically relevant sample (QUADAS items 1, 2).
	● the index test should not form part of the reference standard (item 7).
	● evaluators should be masked to results of index test and reference test (items 10, 11)
	● the tests should be described in sufficient detail to permit replication (items 8, 9).
	● sample size ≥ 5000.
	● diagnostic accuracy presented as sensitivity and specificity.
*Moderate: moderate risk of bias*	Prospective study design
	Since no prospective studies based on digital mammography could be identified, scanned analogue images were accepted. Otherwise the same criteria as for high quality were required.
*Low: high risk of selection and/or verification bias*	Retrospective study design. Selected or enriched samples

### Rating evidence across studies

The quality of the evidence of each method’s/test’s diagnostic accuracy was rated in four levels according to GRADE [22][23]:

· High (⊕⊕⊕⊕). Based on high or moderate quality studies containing no factors that weaken the overall judgement.

· Moderate (⊕⊕⊕O). Based on high or moderate quality studies containing isolated factors that weaken the overall judgement.

· Limited (⊕⊕OO). Based on high or moderate quality studies containing factors that weaken the overall judgement.

· Insufficient (⊕OOO). The evidence base is insufficient when scientific evidence is lacking, the quality of available studies is low or studies of similar quality are contradictory.

Applying GRADE serves to obtain answers to the following questions. How much confidence can one have in a particular estimate of effect? Is the result sustainable, or is it likely that new research findings will change the evidence in the foreseeable future? The rating starts at high, but confidence in the evidence may be reduced for several reasons, including limitations in the study design and/or quality, inconsistency or indirectness of results, imprecise estimates and probability of publication bias. Any disagreements on inclusion/exclusion criteria, rating quality of individual studies or quality of evidence of test methods were solved by consensus.

· Sensitivity = probability that a person with a disease has a positive test result.

· Specificity = probability that a healthy person has a negative test result.

· Relative sensitivity = number of detected cancer cases per reader divided by the total number of detected cancer cases.

· Population based mammography screening = all women in certain age groups receive a personal mailed invitation to get a mammogram at regular intervals (1.5 – 3 years)

· Cancer detection rate = the number of cancer cases detected per 1000 women examined.

· Recall rate = the number of women per 1000 woman recalled for further investigation.

· Interval cancer = cancer cases detected between two screening occasions.

## Results

The results of the literature search and the outcome of the selection procedures are shown in a flow chart (Figure 2).

Fifty-three articles were reviewed in full text. Nine of them were review articles [12,16-18,24-28]. Many studies had not been performed in screening settings or had selected or enriched populations, sometimes without comparison between single reading + CAD and double reading [14,29-57]. Nine studies had large populations, but compared only single reading + CAD with single reading [58-66]. One study that only described different cancer types was excluded [67].

Four studies were included in the summary results, Table 2 (see Additional file 1). Three of them had methodological shortcomings and were judged to be of low quality [68-70]. Only one study, of moderate quality, was included in the GRADE synthesis, Table 3[71]. This was a prospective multicentre study based on the UK national screening program and including 28,204 women aged 50–70 years. No statistically significant difference was found between single reading + CAD and double reading for cancer detection rate (7.02/1000 and 7.06/1 000). The overall agreement between the two strategies was 74.9% (170/227). However, single reading with CAD gave a significantly higher recall rate (3.9% versus 3.4%; p = 0.001). Compared to double reading, single reading with CAD gave lower sensitivity (87.2% versus 87.7%) and lower specificity (96.9% versus 97.4%) but the differences were not statistically significant. Due to incomplete follow-up, sensitivity was likely to be overestimated. Overall, there was no statistically significant difference between the two strategies as regards pathological characteristics of the 57 detected cancers. Study results are reported in Tables 2 and 3. 

**Table 2:** Main characteristics, results and quality rating of four studies on mammography screening

**Author, Year (ref)**	**Study design, Study period,Population, Readers**	**Index test (I)**	**Reference test**	**Results CI= confidence interval Se= sensitivity Sp=specificity**	**Study quality, Comments**
Gilbert et al., 2008 [71]	Prospective, multicentre 2006-2007	I.1: single reading + CAD, n=28,204	Biopsy of suspected cases or follow-up (not all, though; number not reported)	*Cancer detection rate:*	Moderate
				Single reading + CAD: 7.02 /1000.	
	*Population:*			Double reading: 7.06/1000.	Restricted generalisability since results were based on single reading +CAD by experienced radiologists.
				Difference not statistically significant (NS).	
		I.2: double reading, n=28,204.			
	Initially invited: 68,060 women.				
				*Recall rate:*	Incomplete follow-up, particularly affecting the estimates of sensitivity.
	Investigated: 28,204.				
	Aged 50-70 years (1 % > 70 years).			Single reading + CAD: 3.9 %.	
				Double reading: 3.4 %.	Scanned analogue mammograms.
				Difference 0.5 % (95 % CI: 0.3;0.8).	
	*Readers:* radiologists (n=17), specially trained staff (n=10).				
				*Accuracy:*	
				Single reading + CAD:	
				Se= 87.2 %	
				Sp= 96.9 %	
	All readers had at least 6 years’ experience and >5000 readings/year			Double reading:	
				Se= 87.7 %	
				Sp= 97.4 %	
				Difference in sensitivity:	
				0.5 % (95 % CI:	
				-7.4;6.6), (NS).	
				Difference in specificity 0,5% ( CI not specified but reported NS).	
Gromet et al., 2008 [69]	Retrospective	I.1: Single reading + CAD	Biopsy and follow-up	*Cancer detection rate:*	Low
	*Population:*			Single reading + CAD: 4.2/1000.	Retrospective study (controlled for age and time since last screening).
	231 221 women			Double reading: 4.46/1000 (NS).	
	2001-05	n=118,808.			
		I.2: Double reading			Follow-up time unclear.
	*Readers:*				
					Screening situation not applicable to European conditions (i.e. recall rate higher than accepted in Europe).
	Single reading + CAD: specialists in mammography.				
		n=112,413.		*Recall rate:*	
				Single reading + CAD: 10.6 %.	
	Double reading: Specialists in mammography + radiology.			Double reading:11.9%.	
				Difference statistically significant (p=0.001).	
					Invitation procedure and blinded readings unclear.
				*Accuracy:*	
				Single reading + CAD: Se= 90.4 %	Scanned analogue mammograms.
				Double reading:	
				Se=88.0 %.	
				Difference statistically significant.	
				Percent of recalled with cancer:	
				Single reading + CAD: 3.9%.	
				Double reading: 3.7% (NS).	
Georgian-Smith et al., 2007 [68]	Prospective	I.1: Single reading + CAD	Biopsy and at least 12 months´ follow-up to detect false negatives.	*Cancer detection rate:*	Low
	*Study period:* 2001-03			Single reading +CAD: 2.0/1000.	Screening situation not applicable to European conditions. Invitation procedure not described.
		n=6381.		Double reading: 2.4/1000 (NS).	
	*Population:* 6381 consecutive screening examinations				
		I.2: Double reading			
				*Recall rate:*	Population, selection criteria, withdrawals unclear.
		n=6381.		Single reading +CAD: 7.87%.	
				Double reading: 7.93% (NS).	
	*Readers:*				Not independent double reading but blinded to CAD
	Experienced breast radiologists			*Accuracy:*	
				Sensitivity and specificity not reported.	Number of recalls based on all readings.
	Single reading + CAD.				Scanned analogue radiographs.
	Double reading: Not independent reading.				
Khoo et al., 2005 [70]	Prospective	I.1: Single reading +CAD n= 6111.	Biopsy	*Cancer detection rate:*	Low
	*Study period:* not reported.		Not reported	Total for double reading + single reading + symptomatic patients:10/1000.	A so-called relative sensitivity used since 3-year follow-up not yet achieved.
			No follow-up		
	*Population:* 6,111 women (45-94 years), screening every 3rd year				
				Not reported individually for the groups.	
					Relatively high screening age and long screening intervals.
		I.2: Double reading n= 6111.			
				*Recall rate:*	
				Single reading + CAD: 6.1%.	Unclear whether the readings were blinded.
				Double reading: 5.0 %.	Incomplete follow-up.
	*Readers:*			Difference statistically significant	Scanned analogue radiographs.
	Radiologists (n=7) and specially trained staff (n=5).				
				*Accuracy:* (relative sensitivity)*	
				Single reading + CAD: Se= 91.5%.	
				Double reading: Se= 98.4% (NS).	
	Double reading not always performed by two radiologists.				

* Relative sensitivity= number of detected cancer cases per reader divided by all detected cancer cases (due to lack of follow-up).

**Table 3:** **Quality of evidence of the difference between single reading (radiologist plus CAD) and double reading (two radiologists) related to cancer detection rate and recall rate in mammography screening (GRADE). Data from Gilbert et al.**[71]

**Outcome**	**Sample size (no. of studies)**	**True positive: Single reading + CAD (95% CI)**	**True positive: Double reading (95% CI)**	**Absolute difference (95%CI)**	**Quality of evidence**	Rating based on study design/quality, indirectness, consistency, precision and publication bias**
Cancer detection rate	28,204 (1)	0.702%	0.706%	0.004%	(⊕OOO)	Study quality –1
		(0.6–0.8)	(0.6–0.8)	(NS*)	Insufficient	Indirectness–1
Recall rate	28,204 (1)	3,9%	3,4%	0,5%	(⊕OOO)	Study quality –1
		(3,7–4,1)	(3,2–3,6)	(0,3–0,8)	Insufficient	Indirectness -1 One study –1

*NS = no statistically significant difference.

** Study quality = Risk of bias, that is, sensitivity probably overestimated due to incomplete follow-up of women with negative test results.

Indirectness = Only breast radiologists with long clinical experience took part in the study.

Lack of precision = The difference in sensitivity between double reading and single reading + CAD has wide confidence intervals.

Because of their shortcomings, the remaining three studies were not considered in our conclusions. However they deserve to be described. Two were conducted in the U.S.A. [68,69], where population-based screening programs are not used. The populations are less well described and it is not clear whether the women received a personal invitation or had sought to get for mammography on their own. Moreover, recall rates were 8–12%, notably higher than recommended in Sweden and Europe (<5%). The larger of these two studies was retrospective and included 231,221 women who underwent mammography screening [69]. The other study was prospective with 6381 consecutive screening examinations [68]. Their results showed no statistically significant difference in cancer detection rate and the recall rates were inconsistent.

The third study was conducted within the framework of the United Kingdom National Health Service Screening Programme [70]. It was prospective and included 6111 screening examinations with a relatively high total cancer detection rate; 10/1000 including those detected by double reading and single reading with CAD and because of symptoms. Even women over 64 years of age (the upper limit for screening in the UK) were included, which may partly explain the relatively high prevalence of cancer cases. Another explanation may be that the interval between screening sessions was three years (usually 1.5-2 years in Europe). Due to lack of follow-up, the authors calculated a so-called relative sensitivity, where single reading + CAD gave a lower but not statistically significantly different sensitivity of 91.5% compared to 98.4% with double reading. Single reading + CAD had a significantly higher recall rate (6.1%) compared to double reading (5.0%).

To conclude, these three studies show partly conflicting results and it is difficult to draw any conclusions. According to Gilbert et al. [71], the two reading methods resulted in equal numbers of cancer cases. However, this was achieved at the expense of a statistically significantly higher recall rate, implying unnecessary additional examinations. Recall rates in the two studies from the USA [68,69] were two to three times higher than in Sweden (average 3% [20,72]) and not in accordance with European guidelines (<5% [11]).

### Economic aspects

The results of the literature search on economic aspects show that out of 44 abstracts, only one led to the inclusion of the full-text article [14]. The medical scientific evidence was insufficient to study cost-effectiveness and the quality of the study was judged to be low.

## Discussion

The results of this systematic review indicate that the scientific evidence is insufficient to determine whether single mammographic reading by one breast radiologist + CAD is as accurate as the current practice of double reading involving two breast radiologists.

CAD has been developed to act as a second reader for two main reasons: to enhance the diagnostic sensitivity of mammography screening and to compensate for the lack of trained breast radiologists. Most of the literature on CAD for mammography comprises studies concerning technical aspects, such as improvements to software, analysis of subtypes of breast cancer, e.g. microcalcifications only, densities only, distortions or combinations of these. The majority of the clinical studies was performed on selected materials enriched with cancer cases, and thus did not represent a true screening situation. Furthermore, comparison with double reading was not a standard procedure in many of the studies. Since the aim of this review was to critically evaluate the scientific evidence of CAD’s performance in large population-based screening programs, only four studies met our strict inclusion criteria [68-71]. Of these, only one was considered to have sufficient relevance and quality [71].

Two major shortcomings in study design apply to all four included studies. One is survival rate, which is the most important outcome in mammography screening. None of these studies compared the survival rates with the two strategies, and therefore the present outcome measures (cancer detection rate and recall rate) can be regarded as surrogate outcomes. The other shortcoming is incomplete follow-up. As pointed out in the study by Gilbert et al. [71], sensitivity will be overestimated because of this shortcoming.

Although the study by Gilbert et al. [71] comprised a large population and had an elaborate study set-up, its generalisability is limited since all participating breast radiologists had extensive experience of mammography screening. This is not always the case in an authentic setting. The impact of CAD performance on scanned analogue radiographs as compared to digital mammography is also a matter of concern.

Initially, all CAD studies were performed on scanned analogue mammograms that were analysed with CAD. Over time there has been a transition from analogue to digital mammography and this process is still ongoing in many parts of the world. The reliability of CAD analysis of scanned films has been questioned [73]. This aspect, together with the fact that modern mammography is performed in a digital environment, implies that new studies are required to fully understand CAD’s performance and outcomes in large population-based screening programmes using digital mammography.

Lack of trained radiologists remains a problem even when CAD is used. Using CAD as a first/second reader due to unavailability of a trained breast radiologist could be unsustainable, for instance due to retirement. In any case, new generations of breast radiologists must be secured. Besides, being able to discuss uncertain cases with an experienced colleague is absolutely essential, both for educational purposes and in order to avoid too many false positives/false negatives. When working with CAD, a single radiologist will always have to make the final decision to recall or not to recall a woman for further work-up. This decision may depend on a single CAD mark in an area where the radiologist did not react initially. In our opinion, the single radiologist using CAD needs to be highly experienced, particularly when deciding not to recall a woman for further work-up when a potential cancer might be missed. In conclusion, education and training of new generations of breast radiologists have to be done irrespective of the use of CAD, although it has been suggested that CAD could be used in the training of radiologists [74].

As pointed out earlier, screening policies vary between countries and this review has been performed from a European perspective. However, all screening settings have some features in common, be they population-based, centrally-organized or non-organized (“wild” or “opportunistic” screening) mammographies on asymptomatic women. High throughput is one of these factors that place high demands on smooth screening workflows. Integrating CAD into the workflow would mean that the radiologist would have actively to consider all CAD prompts, which in turn increases the total reading time.

High recall rates imply that more women have to return for additional investigation, involving new mammographic images and often also ultrasound examination. In addition, some have to undergo biopsy and in some cases even surgery. This also means more visits to doctors/hospitals for these women. Overall, additional resources are required and women are worried unnecessarily. Since the medical consequences are not convincingly positive, it is not possible to determine either the cost-effectiveness and/or the socioeconomic consequences of replacing one of the readers with CAD in the context of mammography screening.

## Conclusions

The conclusions from this systematic review are:

· The scientific evidence is insufficient to determine whether CAD + *single reading* by one breast radiologist would yield results that are at least equivalent to those obtained in standard practice, i.e. *double reading* where two breast radiologists independently read the mammographic images.

· Since the medical consequences are uncertain, it is not possible to determine the cost-effectiveness or the socioeconomic consequences of replacing one of the readings with CAD in the context of mammography screening.

· Since this literature review, CAD technology has advanced further, thanks to improvements in computer software and digitalization.

· Additional prospective and preferably randomized population-based studies are essential to understand the method’s specific benefits, consequences, and costs.

## Competing interest

The authors declare that they have no competing interest.

## Authors’ contributions

EA: Study concept, analysis, interpretation of data and drafting the manuscript. SZ: Study concept, analysis, interpretation of data and drafting the manuscript. IM: Study concept, analysis, interpretation of data and drafting the manuscript. MHA: Study concept, analysis, interpretation of data and drafting the manuscript. All four authors are responsible for the content and writing of the paper and approved the final manuscript.

## Authors’ information

*First authorship shared by Azavedo E and Zackrisson S.
